# High Na^+^ Salt Diet and Remodeling of Vascular Smooth Muscle and Endothelial Cells

**DOI:** 10.3390/biomedicines9080883

**Published:** 2021-07-24

**Authors:** Ghassan Bkaily, Yanick Simon, Ashley Jazzar, Houssein Najibeddine, Alexandre Normand, Danielle Jacques

**Affiliations:** Department of Immunology and Cell Biology, Faculty of Medicine and Health Sciences, Université de Sherbrooke, Sherbrooke, QC J1H 5N4, Canada; yanick.simon@usherbrooke.ca (Y.S.); ashley.jazzar@usherbrooke.ca (A.J.); houssein.najibeddine@usherbrooke.ca (H.N.); alexandre.normand@usherbrooke.ca (A.N.); danielle.jacques@usherbrooke.ca (D.J.)

**Keywords:** Na^+^ salt, hypertension, Na^+^ salt sensitive hypertension, vascular endothelial cells, vascular smooth muscle cells, glycocalyx, Na/Ca exchanger, Na/H exchanger

## Abstract

Our knowledge on essential hypertension is vast, and its treatment is well known. Not all hypertensives are salt-sensitive. The available evidence suggests that even normotensive individuals are at high cardiovascular risk and lower survival rate, as blood pressure eventually rises later in life with a high salt diet. In addition, little is known about high sodium (Na^+^) salt diet-sensitive hypertension. There is no doubt that direct and indirect Na^+^ transporters, such as the Na/Ca exchanger and the Na/H exchanger, and the Na/K pump could be implicated in the development of high salt-induced hypertension in humans. These mechanisms could be involved following the destruction of the cell membrane glycocalyx and changes in vascular endothelial and smooth muscle cells membranes’ permeability and osmolarity. Thus, it is vital to determine the membrane and intracellular mechanisms implicated in this type of hypertension and its treatment.

## 1. The Vascular System

The vascular system is a closed transport network with a rigid structure that contracts and relaxes. Under the impulse of the cardiac pump, this system ensures the transport of blood to supply the cells with oxygen and necessary nutrients and to eliminate their waste products for their proper functioning and the maintenance of their homeostasis [1]. 

**Figure 1:**
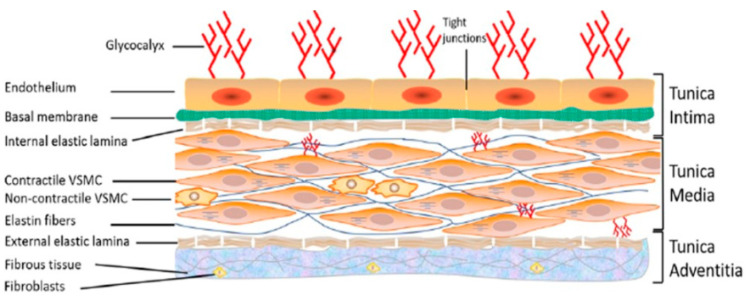
Structure of the vascular wall. Schematic representation showing the three layers of the vascular wall: tunica intima, tunica media, and tunica adventitia, as well as the components of each layer. VSMC: vascular smooth muscle cell. From Bkaily et al., 2021 [2].

From a macroscopic perspective, this system is divided into three major categories: veins (afferent vessels operating in a low-pressure system), arteries (efferent blood vessels operating in a high-pressure system), and capillaries (connecting veins and arteries). Microscopically, except the capillaries, artery and vein histological organization and their tissue composition are similar and divided into three major tunics or layers (Figure 1) [2].

On the surface, there is the tunica externa or adventitia, which is composed of loosely intertwined collagen fibers and fibroblasts (Figure 1) [2]. Between the circulating blood and the vascular wall is the tunica interna or intima, consisting primarily of a monolayer of vascular endothelial cells (VECs) (Figure 1) [2]. Finally, between the intima and the adventitia lies the tunica media or media composed of abundant elastic fibers and particularly contractile vascular smooth muscle cells (VSMCs), which control the vessel tone (Figure 1). VECs and VSMCs are heterogeneous. VSMCs are a fusiform cell population of approximately 200 μm in length and 5 μm in diameter. However, VECs are roughly 30–50 µm in length, 10–30 µm wide, and a thickness of 0.1–10 µm. Both cell types are of multiple embryonic origins (neural crests, mesoderm) [2,3].

**Figure 2:**
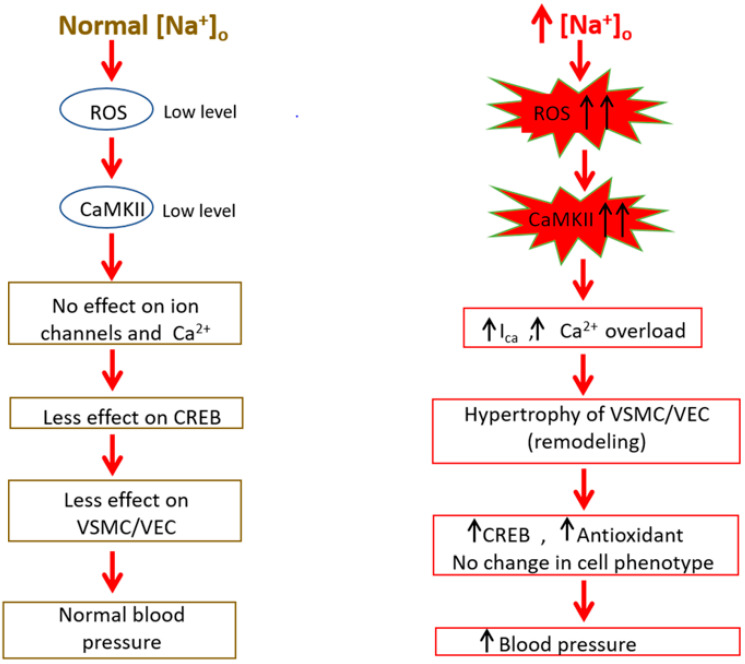
Schematic representation summarizing the literature in the field showing that chronic high salt induced an increase in the intracellular levels of ROS (reactive oxygen species) and activation of CaMKII (calmodulin kinase II) and cyclic AMP response element binding protein (CREB). I_Ca_: L-type Ca^2+^ channels; [Na^+^]_o:_ extracellular Na^+^ concentration.

**Figure 3:**
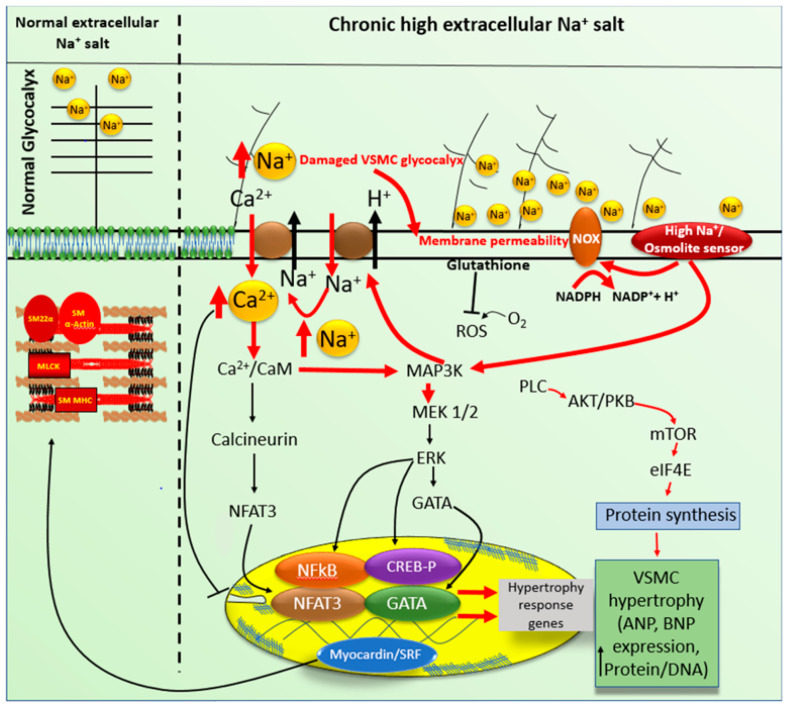
Schematic representation summarizing the literature on the glycocalyx, plasma membrane Na^+^ transport, and Ca^2+^-dependent signaling and transcription factors that could be implicated in chronic high salt induced vascular smooth muscle and endothelial cells. ROS: reactive oxygen species; Ca^2+^/CaM: calcium calmodulin; VSMC: vascular smooth muscle cells. Modified from Bkaily et al., 2021 [2].

Due to the complexity of their origin during early embryogenesis, understanding the differentiation of these cells remains a significant challenge [2,4]. Under physiological conditions, contractile VSMCs in mature blood vessels exhibited a low rate of synthesis of extracellular matrix components and was characterized by a series of highly regulated smooth muscle markers, such as cytoskeletal and contractile proteins, which include smooth muscle actin, myosin heavy chain, calponin, and smooth muscle 22 alpha (SM22α), as well as signaling molecules [2,4,5,6]. All are required for the primary function of VSMCs [4,7]. However, because contractile proteins and several transcription factors are Ca^2+^ dependents, the contractility of VSMCs is determined primarily by intracellular Ca^2+^. Therefore, to regulate the various Ca^2+^-dependent functions under normal conditions and during excitation-contraction coupling, VSMCs use various ion transporters and must keep intracellular Na^+^ at a low concentration [8,9]. VECs markers are numerous, but the most used is the von Willebrand factor. They do not possess L-type Ca^2+^ channels, and the Ca^2+^ influx takes place via the R-type Ca^2+^ channels [10] and the Na/Ca exchanger. In both cell types, Na^+^ influx takes place mainly via the Na/Ca and Na/H exchangers (Figure 2 and Figure 3). These two membrane exchangers must effectively control intracellular Ca^2+^ and Na^+^ homeostasis [5,6].

## 2. Vascular Remodeling

The concept of vascular remodeling was first described by Baumbach, based on observations in arterioles of hypertensive rats [11]. It is an active process of structural adaptation of VSMCs and VECs to hemodynamic changes or to long-term vascular damage [12]. Depending on the type of hemodynamic changes and VECs and/or VSMCs injury, vascular remodeling is characterized structurally by hypertrophy (wall thickening), eutrophy (constant wall thickness), or hypotrophy (wall thinning) [12,13,14,15]. These structural changes may be eccentric (increased remodeled arterial lumen) to accommodate reduced luminal space due to atherogenic lesions or post-intraluminal restenosis, to maintain adequate blood flow [14]. However, these changes may be concentric (reduction in remodeled arterial lumen) due to prolonged wall tension and vasoconstriction as in hypertension [14,16,17].

Among the different cellular and molecular mechanisms observed, the media is the most active layer [18]. As a result, VSMCs contribute largely to the phenomenon of vascular remodeling by undergoing morphological changes characterized mainly by hypertrophy and hyperplasia [19] without changes in the contractile phenotype of the cells [2]. Little is known about the structural and morphological remodeling of VECs and, more particularly, in humans.

As all muscle cells, VSMCs also undergo hypertrophy in response to local stimuli [20,21,22]. This hypertrophic process is a cell growth response characterized by increased cell size with or without an increase in protein synthesis or changes in the cell phenotype [2,20,23]. In general, this cell remodeling may be due, on the one hand, to physiological conditions as in pregnancy [24] or secondary to physical exercise [25]. On the other hand, it can be due to pathological conditions, such as hypertension [26] and high sodium salt diet [20]. During these pathologies, an elevation of intracellular Ca^2+^ level of VSMCs and an increase in sensitivity to vasoactive stimuli have been reported [20,27,28,29]. These pieces of information indicate that hypertrophied VSMCs, similar to cells with a contractile phenotype, retain the ability to interact with stimuli and increase intracellular Ca^2+^ levels following a signaling pathway different from normal calcium dynamics [30,31]. Indeed, the mechanism underlying VSMCs hypertrophy is poorly studied and may occur due to the degradation of protein inhibitors or protein synthesis stimulation [19,32]. Atef and Anand-Srivastava, including other authors, have demonstrated that the Gqα protein-related signaling pathway is a classical pathway of the hypertrophic response of VSMCs [33,34]. Generally, this pathway can be triggered by growth factors, or by one or more varieties of vasoactive substances, including angiotensin II, endothelin-1and neuropeptide Y [19,21,32,35]. 

## 3. Sodium and Sodium Transport in Vascular Smooth Muscle and Endothelial Cells

Intracellular free Ca^2+^ and Na^+^ are not homogeneously distributed in excitable and non-excitable cells [36,37,38]. When VSMCs or VECs are at rest, the concentration of free Ca^2+^ in the cytoplasm, perinucleoplasm, and nucleoplasm are 50, 600, and 300 mmol/L, respectively [36,37]. Furthermore, the concentration of Na^+^ in these three compartments is 10, 40, and 20 mmol/L, respectively [10,36]. As in resting cells, in response to increased Na^+^ or Ca^2+^ influxes across the sarcolemma membrane, the concentration of nucleoplasmic free Ca^2+^ and Na^+^ are higher than that of the cytoplasm and lower than that of the perinucleoplasm [10,20,36,38].

As mentioned previously, in VSMCs and VECs, there are several types of Na^+^ transporters: the Na^+^/H^+^ exchanger (NHE1), the Na^+^/Ca^2+^ exchanger (NCX) and the Na^+^/K^+^ pump (Figure 2 and Figure 3). Functionally, NHE-1 is involved in cytoskeletal organization, cell volume regulation [39], differentiation, proliferation [40,41], cell migration, and even apoptosis [42]. It plays a primary role in the pH regulation at both the cytosolic and nuclear levels [38,43,44]. In the nucleus, it is known to be involved in the activation of chromatin [45] and nuclear pore functioning [38,46]. Undoubtedly, this exchanger’s activity can directly affect gene expression and perinucleoplasmic, nucleoplasmic, and cytoplasmic homeostasis of Na^+^ and Ca^2+^ under normal and pathological conditions [37,38,43]. During intracellular acidosis, the H^+^ outflux will induce Na^+^ influx through this exchanger and contribute to an intracellular increase of Na^+^ (Figure 2 and Figure 3) [2,20,47]. 

Another important Na^+^ transporter that continually cross-talk with the Na^+^/H^+^ exchanger is the sodium/calcium exchanger isoform 1 (NCX1) (Figure 2 and Figure 3). The NCX1 is a transmembrane protein that was cloned in 1990 and is expressed at the cytoplasmic membrane, nuclear membrane, and in the mitochondria of a variety of cells, such as hepatocytes, cardiomyocytes, endothelial cells, VECs, and VSMCs [37,43,48,49,50,51]. It has an amino-terminal portion composed of 5 transmembrane domains and a carboxy-terminal portion consisting of 4 transmembrane domains [49,50]. These portions are separated by a large cytosolic loop containing an endogenous XIP (exchanger inhibitor peptide) region, a binding site for Ca^2+^ regulation, and a region where alternative splicing occurs [50,51,52]. 

Functionally, this exchanger involves at least 1Ca^2+^ for 3Na^+^ [48,53]. Depending on the Na^+^ concentration gradient, this system can also be reversed [48,53]. In the presence of intracellular Ca^+^ overload and the absence of an increase of intracellular Na^+^, this exchanger excludes Ca^2+^ from the cell [54]. Thus, it regulates Na^+^ homeostasis and indirectly Ca^2+^ homeostasis at the cytosolic, perinucleoplasmic, and nucleoplasmic levels [43]. 

In sum, these two ion transporters are essential for Na^+^ and Ca^2+^ homeostasis [36,43]. At the cytosolic level, this homeostasis is directly regulated by the nucleus [36,55]. Furthermore, independent of the cytosol and like a cell within a cell, the nucleus is regulated by an auto nuclear mechanism that protects it from trauma or damage [36,43,55]. However, an alteration in the cytosolic and/or nuclear compartment of Na^+^ and Ca^2+^ homeostasis could affect excitation-contraction (VSMCs) and excitation-secretion (VECs) coupling, cell function, and survival resulting in vascular remodeling [37,38]. 

There is several other Na^+^ transporters that are less known in VECs and VSMCs, such as the Na^+^-bicarbonate (NBCn), the Na^+^-Cl^−^- K^+^ (NKCC), and the taurine-Na^+^ symporters.

Recently, several reports suggest the presence of an epithelial Na^+^ channel in renal vessels from male C57BL/6J mice [56] and rat mesenteric VSMCs [57,58], as well as in rat mesenteric artery endothelial cells [58,59]. However, this type of channel in VSMCs and VECs, and, more particularly, from the healthy human origin, is still a matter of debate because of the absence of a specific blocker of this channel.

## 4. High Sodium Salt-Induced Salt-Sensitive Memory

Extracellular Na^+^ is a primary determinant of plasma osmolarity and VSMCs tone [60,61,62]. It is finely maintained under normal conditions and in humans at a concentration between 135 and 145 mmol/L [60]. However, the latter depends in particular on the daily salt intake estimated at less than five gr per day (Na^+^: 2400 and Cl^−^: 3000) [63,64,65]. However, in humans, the physiological salt requirement is less than 1 gr per day (Na^+^: 400 and Cl^−^: 600) [66,67,68]. 

With salt as a preservative and to improve the organoleptic character of foods, humans have dramatically increased their consumption to over 9.6 gr per day (Na^+^: 3840 and Cl^−^: 5760) [69,70]. Studies by Chauveau et al. on the proportion of average daily salt consumption in industrialized countries showed that 75% of the salt consumed is found in preserved foods, 15% is related to kitchen preparation, and only 10% is naturally present in foods [69]. This excess consumed salt cannot be eliminated by the kidneys. It can lead to an accumulation of extracellular Na^+^ ([Na^+^]_o_) of 2 to 4 mmol/L [63,65,68,71,72,73,74]. 

It is well known that Na^+^ can have a direct effect on blood pressure [59,62,75,76]. Studies in hypertensive patients have shown that [Na^+^]_o_ increases by 1 to 3 mmol/L [63,77]. According to Suckling and colleagues, ingestion of 6 gr per day of salt in a healthy subject can increase extracellular Na^+^ of 2 mmol/L and osmolarity of 4 mosm/L [78]. Other studies in healthy subjects have shown that an increase in extracellular Na^+^ of 3 mmol/L can lead to alteration of ion transporters and movement of fluid from intracellular to extracellular space associated with secretory excitation of certain hormones, such as aldosterone, renin, and vasopressin [63,73,77]. Changes of only 1% in plasma osmolarity are sufficient to cause a significant increase in plasma vasopressin [79]. The latter can increase blood pressure following tonus contraction of VSMCs [80]. By causing an alteration in intracellular ion homeostasis, prolonged accumulation of extracellular Na^+^ may also promote cellular hyperosmotic stress and contribute to salt-sensitive hypertension [63,80,81]. 

Kawasaki et al. and Weinberger et al. were among the first to recognize the heterogeneity of the blood pressure response to a sodium-rich diet and to develop the concept of salt sensitivity in humans [82,83]. According to clinical findings, it is defined as a factor contributing to an increase in blood pressure of at least 10% [72]. According to De la Sierra and colleagues, salt sensitivity contributes to the rise in mean pressure of more than four mmHg (24-h ambulatory blood pressure monitoring) [84]. Depending on the definitions and measurement methods used, salt sensitivity is observed in 25% to 50% of normotensive subjects (BP < 120/80) and 40% to 75% of hypertensive patients [85]. However, this prevalence appears to be more pronounced in elderly, obese, renal failure, and African American subjects [71].

To date, the mechanism responsible for salt sensitivity remains controversial [63,86]. For a long time, and even today, some authors attribute it to renal malfunction [63,87]. According to them, after a regular hypersodium diet, these salt-sensitive patients show decreased renal blood flow, an increase in renal vascular resistance, and intraglomerular pressure [88]. According to Kawasaki, the inability of the kidneys to excrete excess sodium may be either secondary to primary hyperaldosteronism or renal pathology or genetic [63,82,83,85]. Recently, attention is no longer directed solely to the kidneys but primarily to the vascular system [59,62,65,81,89,90]. Thus, particular interest has been focused on the first barrier located on the surface of the endothelium and vascular smooth muscle cells, the glycocalyx [2,74,91,92]. 

The VSMCs and VECs glycocalyx (Figure 1) is a negatively charged anionic biopolymer layer at hundreds of nanometer thicknesses [68,93]. This thin layer in VECs acts as a barrier and prevents nonspecific adhesion of circulating blood cells to the endothelium, slows blood flow in the capillary system [94] and selectively controls VECs and VSMCs cell membrane Na^+^ permeability and vascular permeability [68,95]. When this layer is exposed to a chronic concentration of 5% NaCl above the standard physiological value, a significant reduction of negatively charged heparan sulfate residues occurs [96]. The loss of these surface charges renders VECs and VSMCs (second protective barrier) vulnerable to unwanted intruders, including excessive sodium, leading to VECs shrinkage to more than 25% [74,91,92]. This may promote increased vascular permeability, the release of VECs vasoactive substances (angiotensin II, endothelin-1) [81,89], which lead to direct exposure of VSMCs to these substances and sodium overload. Such changes in VSMCs extracellular space induce remodeling associated with an increase in muscle tension which leads to hypertension. 

According to various epidemiological studies and animal models developed and used, it is well established that dietary salt intake is the most common and important risk factor for developing essential hypertension [56,59,81,82,89]. It is also well known that a chronic concentration of 2 to 4 mmol/L extracellular Na^+^ can directly affect blood pressure in both normal and hypertensive subjects [63]. However, the cellular and molecular mechanisms underlying salt sensitivity and associated vascular disorders are not fully understood [81]. Some suggest renal dysfunction, while others hypothesize vascular dysfunction by demonstrating impairment of both vascular barriers (endothelial glycocalyx and endothelium) following chronic exposure above 145 mM NaCl [74,91,92].

Knowing that VSMCs are the central contracting cells of the vascular wall, this slight increase in plasma sodium may lead to altered intracellular Na^+^ and Ca^2+^ homeostasis promoting hypertrophy and/or hyperplasia of these cells (Figure 3) [2,97,98,99]. Eventually, this would promote increased peripheral resistance and blood pressure (Figure 3) [62,91]. However, the mechanisms responsible for the increase in blood pressure still obscure.

## 5. High Sodium Salt-Induced VSMCs and VECs Stress

Hyperosmotic stress is an often-overlooked process that potentially contributes to the pathogenesis and progression of various human pathologies (hypertension, diabetes, atherosclerosis, and other cardiovascular diseases) [80]. It is the increase in extracellular osmolarity above the average physiological value (280–300 mOsm/kg H_2_O) [100] that can be observed in various cell types, such as T and B cells, macrophages, neurons, epithelial cells, renal cells, myoblasts, fibroblasts, and, especially, VSMCs and VECs (Figure 2 and Figure 3) [101,102,103]. 

Depending on the cell type, responses to hyperosmotic stress can be variable, and the signaling pathways involved differ from cell to cell [80].

However, in all cell types, hyperosmotic stress is characterized by shrinkage of cell volume, increased oxidative stress (Figure 3) [100], protein carbonylation, mitochondrial depolarization, DNA double-strand breaks caused mainly by activation of p53 and/or p38, and cell cycle arrest [80,104,105]. Depending on the duration of exposure and NaCl concentration, cell cycle arrest is short-lived to prevent cell death by apoptosis and give the cell time to adapt to the increased osmolarity [105]. To date, there are very few studies that specify the different signaling pathways involved [80]. Studies have shown activation of the mitogen-activated protein kinase (MAP kinase) and c-Jun N-terminal kinases (JNK pathway) (Figure 2 and Figure 3) [104,105], including expression of the aquaporin 1 (AQP1) and aquaporin 5 (AQP5) genes, facilitate water movement [106,107]. Besides, increased calcium influx leading to nuclear factor of activated T-cells 5 (NFAT5) activation and nuclear translocation has been observed (Figure 2 and Figure 3) [101,104,105]. The latter leads to the subsequent regulation of target genes, including those associated with osmolyte transport and synthesis, antioxidant defense, and numerous molecular chaperones [80] (Figure 2 and Figure 3).

## 6. High Na^+^ Salt-Induced Glycocalyx Remodeling

Several reviews are available concerning glycocalyx and main, particularly in VEC [108]. In 1940, the cell biologist James Danielli (who discovered that the membrane is a lipid bilayer) hypothesized that a layer of proteins covered the vascular system’s inner walls [108,109]. Later on, this plasma membrane layer was given the name glycocalyx [108,110]. Several groups have highlighted the physiological role of the endothelial glycocalyx, given the importance of this structure as a shear stress sensor of blood flow, thus contributing to blood pressure regulation [58,68,95].

The glycocalyx is formed by two essential components: proteoglycans, syndecans, glypicans, and glycoproteins. [108]. The transmembrane proteoglycans are the critical element in all cell types and, more particularly, in the endothelial glycocalyx. There are four known syndecans in vertebrates: syndecans 1, 2, 3, and 4; however, the glycocalyx endothelial contains primarily syndecan-1 [108].

The glycocalyx also plays a role in mechanotransduction. Since glycocalyx is negatively charged, it acts to buffer positively charged substances and, more particularly, Na^+^, the most abundant positively charged ion. Thus, it contributes to the modulation of extracellular surface charge and acts as a buffer of extracellular Na^+,^ and controls its cell membrane permeability. However, chronic loading of the glycocalyx with Na^+^ at the heparan sulfate residues leads to collapse [110] and a decrease of glycocalyx [20]. Such destruction of the glycocalyx affects plasma membrane charges, which will affect the level of membrane potential, ligand-receptor binding, and ionic transporters. Therefore, damaging the glycocalyx affects the excitation-secretion coupling of VECs [101,108] and excitation-contraction coupling of VSMCs, as well as intracellular homeostasis of Ca^2+^, Na^+^, and ROS (Figure 3) [20]. A deterioration of the glycocalyx seems to occur during aging [111,112,113]. This particular aspect needs to be verified in healthy humans to determine whether this age-dependent deterioration of the glycocalyx is independent of renal dysfunction. Besides, it is not clear in the literature whether the vascular remodeling in a chronic high salt diet is due totally to deterioration and/or decrease in glycocalyx is the main contributor to the physiopathology of chronic high salt diet.

## 7. Adaptive Responses to High Sodium Salt Induced VSMCs Hypertrophy

In response to hyperosmotic stress, cells develop several compensatory and adaptive mechanisms [80,114]. When a small perturbation in extracellular osmolarity occurs, an accumulation of inorganic osmolytes (K^+^, Cl^−^, Na^+^) increases cell volume [115,116,117]. During this process, an increase in NHE-1 activation and an increase in sodium influx have been observed in some studies [105,115]. The increase of ion transporters, in particular for Na^+^, constitutes a double-edged sword by preventing cell volume shrinkage, on the one hand, and severely disrupting intracellular ion homeostasis, on the other hand (Figure 2 and Figure 3) [80]. Secondly, this will lead to an overexpression of genes (SLC2A4, SLC5A3, SLC6A8, SLC9A1) involved in the synthesis (Figure 3) and transport of compatible organic osmolytes (betaine, sorbitol, taurine, choline, creatine, myoinositol, glucose) [80,118,119]. The latter are small molecules concentrated inside the cell that usually have cytoprotective properties, such as antioxidation, and structural stabilization of proteins by acting as chemical chaperones [80,119]. In general, these compatible osmolytes utilize the Na^+^ gradient across the plasma membrane as an electromotive force (Figure 3) [117]. In addition, high extracellular activate an osmolyte sensor, which stimulates NOXs and induces ROS generation (Figure 2 and Figure 3). Taurine is known to stimulate protein synthesis, promote gene expression, and promote increased sodium and calcium influx [2,54,118,119]. Osmolyte accumulation promotes cytoskeletal rearrangement, an essential adaptation in response to increased extracellular osmolarity by allowing the cell to maintain its volume and enhance its structural integrity [80,119]. 

Despite the various adaptive mechanisms, cells adapted to hyperosmotic stress differ from normal cells [105]. They can remodel by hypertrophying [2,20,115] and or proliferating. According to various studies, they enter a state where several changes are associated with multiple persistent lesions, such as DNA double-strand breaks, oxidation of DNA bases and proteins, and cytoskeleton remodeling [80,105]. The mechanism implicated in high salt-induced VSMCs hypertrophy remains to be explored in VECs.

## 8. Na^+^ Salt-Sensitive Hypertension

The role of Na^+^ in the physiological evolution of animals is nicely reviewed by Natochin in 2007 [120]. The relation between high Na^+^ salt consummation and hypertension was first reported in 1904 by Ambard and Beaujard [121,122] and then confirmed by the groups of Dahl [3,120] and Freis [123]. 

Hypertension is among the most critical public health problems worldwide despite advances in prevention, detection, treatment, and blood pressure control [124]. Its prevalence depends on the study population’s racial composition and its criteria. It also depends on sex, gender, and age. In general, it is defined as systolic blood pressure (SBP) > 140 mmHg and/or diastolic blood pressure (DBP) > 90 mmHg at rest 9 stage 2 and on multiple occasions [124,125]. It is a multifactorial disease clinically classified as stage 1 to 3, depending on the severity of blood pressure [124,126]. From the etiological point of view, it is classified into secondary arterial hypertension and essential or primary arterial hypertension. However, in response to salt intake, it has been observed that hypertensive subjects can be salt-resistant (increase in blood pressure less than 10% after salt intake) or salt-sensitive (an increase of BP more than 10% after salt intake) [82,83]. 

Intracellular Na^+^ homeostasis in VEC and VSMC depends partly on the level of plasma circulating Na^+^ [64,127] and on the type and density of plasma membrane Na^+^ transporters. In humans, the physiological need for sodium salts should not exceed 2.5 g per day [68]. Today, salt overconsumption is a significant health issue [127]. Occasional consumption of high Na^+^ salt by healthy humans has no significant effect on blood pressure since the kidneys eliminate it. However, due to the limited capacity of the kidneys to eliminate chronic high Na^+^, the excess of this ion accumulates in the circulation leading to the development of Na^+^ salt-sensitive hypertension [58,110,127,128]. The accumulation of circulating Na^+^ is aggravated in the presence of renal dysfunction [127], with chronic levels reportedly elevated by 2–4 mM beyond normal resting values [63,127]. This chronic increase in circulating Na^+^ was reported to affect only VECs [80] and damages their glycocalyx, leading to remodeling of VECs [20,68,110,128,129,130,131]. However, an increase in vascular permeability will allow VSMC interstitial Na^+^ overload, affecting VSMC glycocalyx (Figure 2 and Figure 3). Our group recently reported the latter aspect to occur together with morphological remodeling and an increase in basal intracellular Na^+^ and Ca^2+^ (Figure 2 and Figure 3). Such a remodeling of VSMCs may lead to hypertension [63,78,83,132,133]. The chronic increase in Na^+^ salts promotes epigenetic ‘salt memory’ programming [110,134], which predisposes the patient to Na^+^ salt-sensitive hypertension [110,127]. This salt memory programming is transmitted to by the parents to their children. Thus, chronic high salt my induce permanent remodeling at the gene level. What is gene implicated? This still to be clarified.

## 9. Implication of ROS/RNS in Na^+^ Sensitive Hypertension

Several review papers highlighted the role of reactive oxygen species (ROS) and reactive nitrogen species (RNS) in the development of several pathologies [135,136,137,138], including hypertension [135]. Cellular radicals (hydroxyl, nitric oxide, nitrogen dioxide and superoxide anion) and nonradicals (hydrogen peroxide, hypochlorous acid, and peroxynitrite) are generated by mechanisms present in the cell, such as: the plasma membrane, the endoplasmic reticulum, the mitochondria, the peroxisomes, and the cytosol [135,136], as well as the nuclear envelop membranes’ and the nucleoplasm [37,38,139]. Na^+^ sensitive hypertension also seems to implicate oxidative stress in many animal models [138,140,141]. This increase in oxidative stress was attributed to inflammation, as well as to renal epithelial cell damage, by activation of NADPH oxidase [136,138,140,142] NOX2/NOX4-derived ROS [138,142]. Such an increase in oxidative stress was reported to activate nuclear factor-kappa B [141], as well as several mechanisms implicated in cell membrane ionic transporters, in addition to aquaporin 1 sensitive transmembrane transporter of hydrogen peroxide [136,143,144] and osmolyte sensor (Figure 3). However, recent literature in the field showed that high -salt-sensitive hypertension is due, at least in part, to damage of the glycocalyx of both vascular endothelial [136] and vascular smooth muscle [20] cells. It is logical to mention that sustained high salt would first induce damage to endothelial cells, and then the inflammation would follow. It is worth noting that the effect of high salt could be relatively less important in one particular vascular cell type compared to another due to the relative density and presence of different types of NOXs, as well as to the different basal levels of oxidant and anti-oxidant factors [20]. Although the mitochondria play an essential role in ROS generation, we should not forget that the nucleus may also contribute to the ROS generation in high-salt sensitive hypertension via probably activation of the calcium-dependent NOX5 [139]. Thus, it is imperative to revisit the nature of essential ROS/RNS generation in high-salt sensitive hypertension and, more particularly, in human vascular endothelial and smooth muscle cells.

## 10. Conclusions

Although Na^+^ sensitive hypertension was reported for the first time in 1904, our knowledge of this disease is still limited and, there is no yet cure for such vascular illness yet. In addition, the mechanisms implicated in developing a memory of high Na^+^ salt-sensitive hypertension is still obscure. There still a lot to be done in this field that is to be rediscovered. Several questions need to be answered: is the damage to the glycocalyx the main most important factor contributing to the development of hypertension and Na^+^ salt sensitivity? Is the chronic increase of extracellular osmolarity induced permanent remodeling of VECs and/or VSMCs? What is the mechanism implicated in the development of salt-sensitive hypertension? To answer these questions and other questions, we need to develop more representee in vivo and/or in vitro models that help us better explore this type of hypertension and develop specific treatment.
